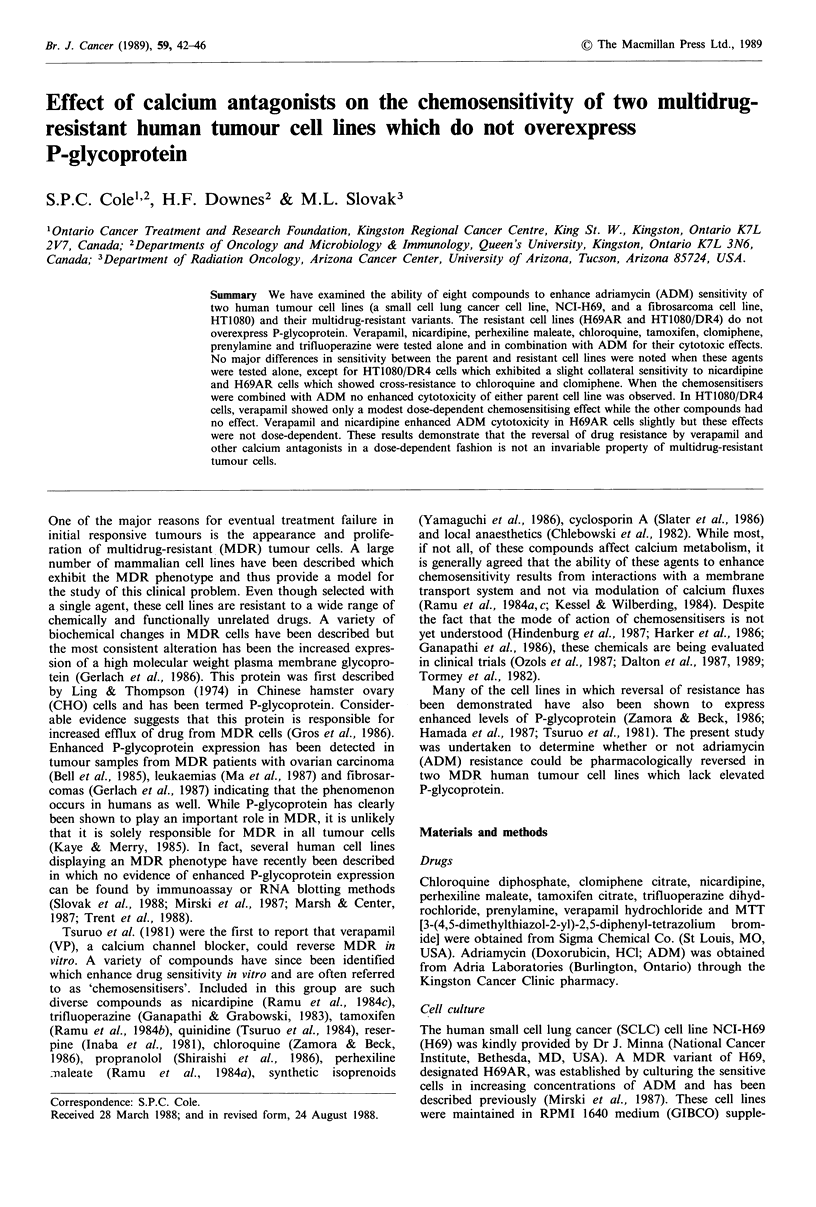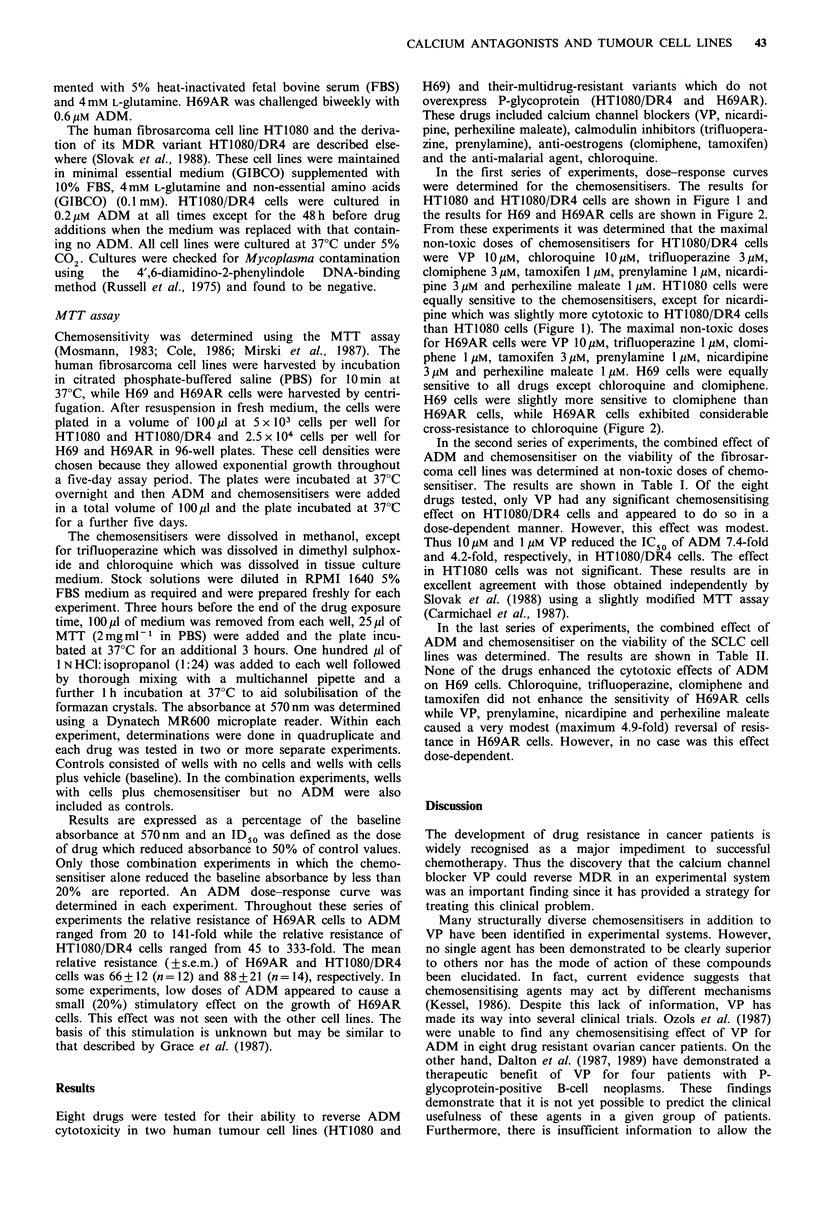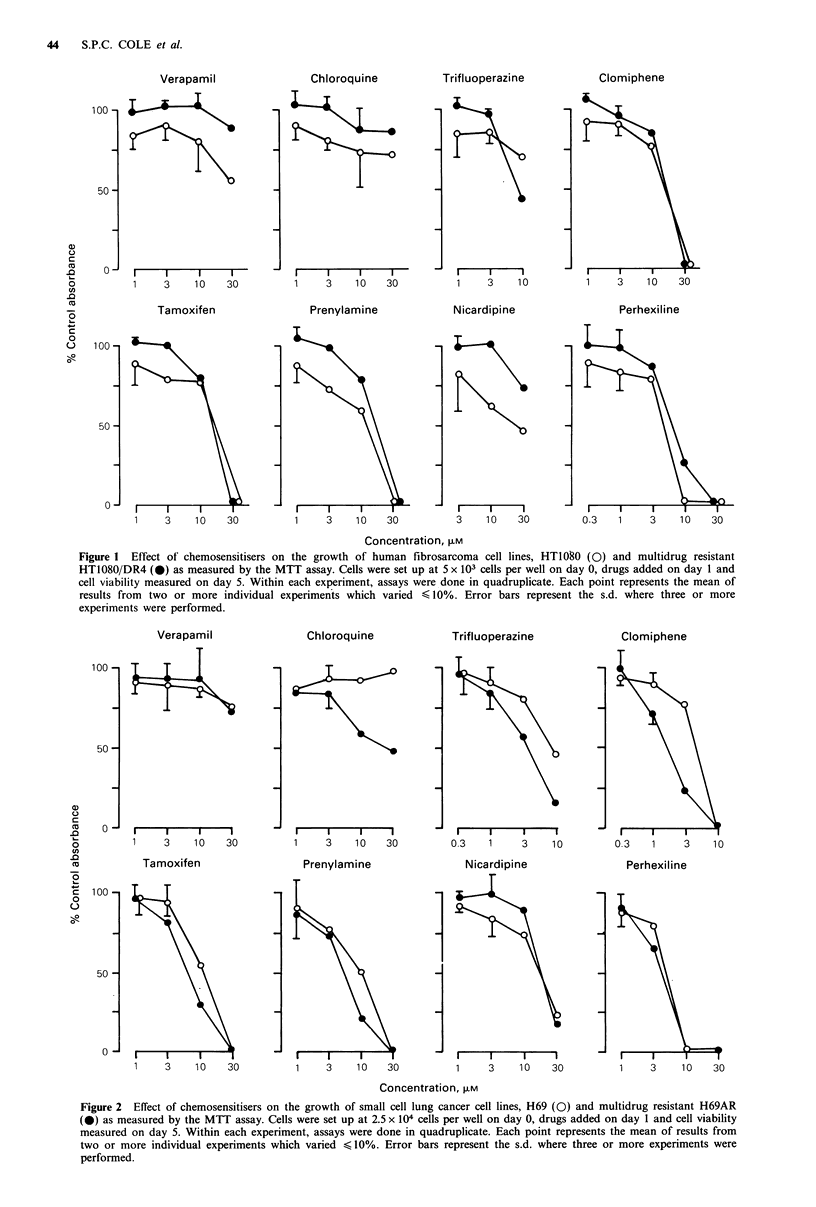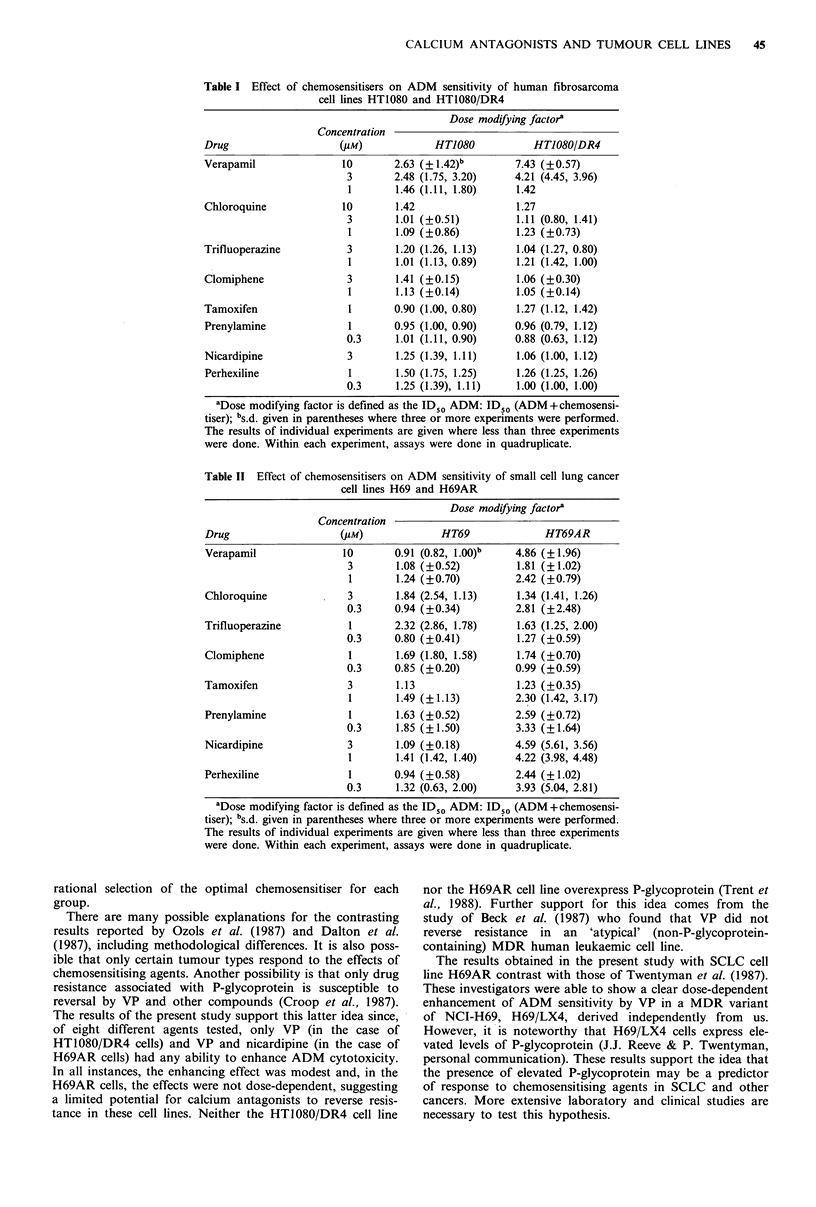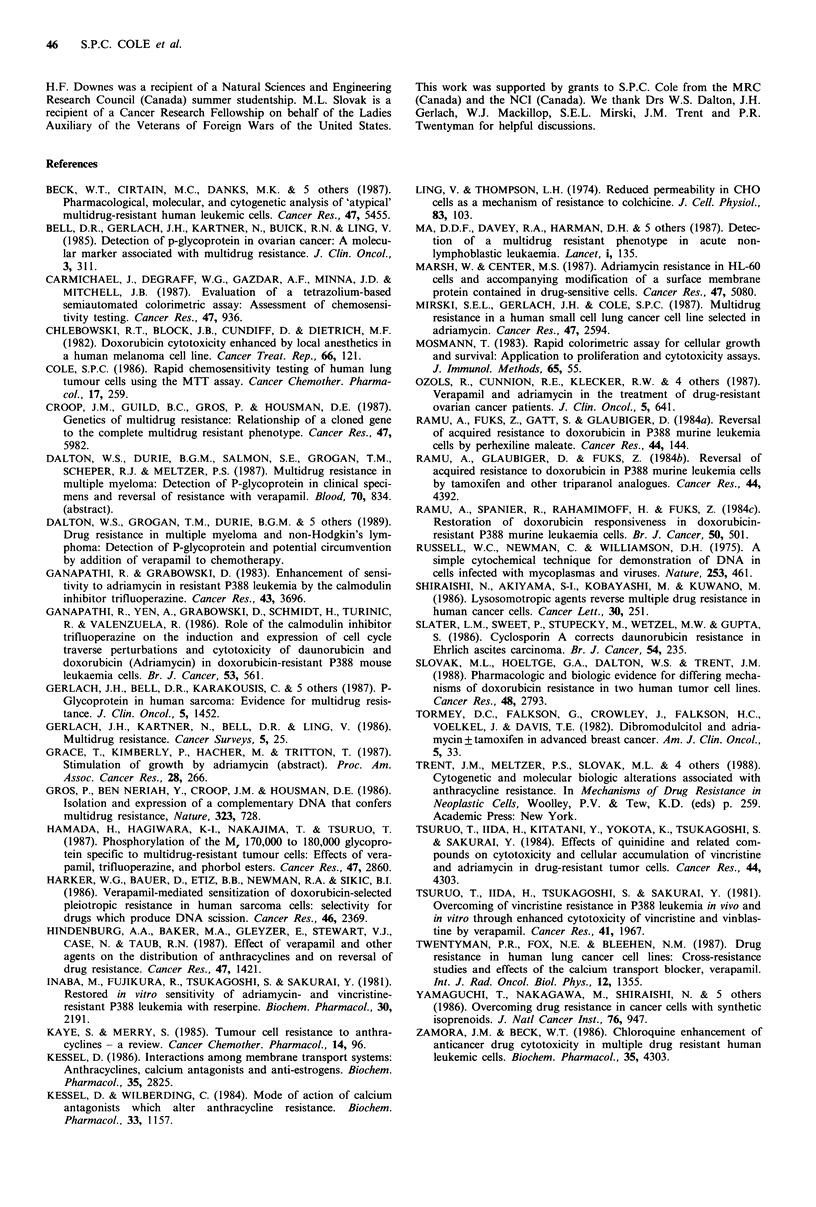# Effect of calcium antagonists on the chemosensitivity of two multidrug-resistant human tumour cell lines which do not overexpress P-glycoprotein.

**DOI:** 10.1038/bjc.1989.9

**Published:** 1989-01

**Authors:** S. P. Cole, H. F. Downes, M. L. Slovak

**Affiliations:** Ontario Cancer Treatment and Research Foundation, Kingston Regional Cancer Centre, Canada.

## Abstract

We have examined the ability of eight compounds to enhance adriamycin (ADM) sensitivity of two human tumour cell lines (a small cell lung cancer cell line, NCI-H69, and a fibrosarcoma cell line, HT1080) and their multidrug-resistant variants. The resistant cell lines (H69AR and HT1080/DR4) do not overexpress P-glycoprotein. Verapamil, nicardipine, perhexiline maleate, chloroquine, tamoxifen, clomiphene, prenylamine and trifluoperazine were tested alone and in combination with ADM for their cytotoxic effects. No major differences in sensitivity between the parent and resistant cell lines were noted when these agents were tested alone, except for HT1080/DR4 cells which exhibited a slight collateral sensitivity to nicardipine and H69AR cells which showed cross-resistance to chloroquine and clomiphene. When the chemosensitisers were combined with ADM no enhanced cytotoxicity of either parent cell line was observed. In HT1080/DR4 cells, verapamil showed only a modest dose-dependent chemosensitising effect while the other compounds had no effect. Verapamil and nicardipine enhanced ADM cytotoxicity in H69AR cells slightly but these effects were not dose-dependent. These results demonstrate that the reversal of drug resistance by verapamil and other calcium antagonists in a dose-dependent fashion is not an invariable property of multidrug-resistant tumour cells.


Br. J. Cancer (1989), 59, 42–46 © The Macmillan Press Ltd., 1989

### Effect of calcium antagonists on the chemosensitivity of two multidrug-resistant human tumour cell lines which do not overexpress P-glycoprotein

S.P.C. Cole[1,2], H.F. Downes[2] & M.L. Slovak[3]

[1]*Ontario Cancer Treatment and Research Foundation, Kingston Regional Cancer Centre, King St. W., Kingston, Ontario K7L 2V7, Canada;* [2]*Departments of Oncology and Microbiology & Immunology, Queen's University, Kingston, Ontario K7L 3N6, Canada;* [3]*Department of Radiation Oncology, Arizona Cancer Center, University of Arizona, Tucson, Arizona 85724, USA.*

**Summary** We have examined the ability of eight compounds to enhance adriamycin (ADM) sensitivity of two human tumour cell lines (a small cell lung cancer cell line, NCI-H69, and a fibrosarcoma cell line, HT1080) and their multidrug-resistant variants. The resistant cell lines (H69AR and HT1080/DR4) do not overexpress P-glycoprotein. Verapamil, nicardipine, perhexiline maleate, chloroquine, tamoxifen, clomiphene, prenylamine and trifluoperazine were tested alone and in combination with ADM for their cytotoxic effects. No major differences in sensitivity between the parent and resistant cell lines were noted when these agents were tested alone, except for HT1080/DR4 cells which exhibited a slight collateral sensitivity to nicardipine and H69AR cells which showed cross-resistance to chloroquine and clomiphene. When the chemosensitisers were combined with ADM no enhanced cytotoxicity of either parent cell line was observed. In HT1080/DR4 cells, verapamil showed only a modest dose-dependent chemosensitising effect while the other compounds had no effect. Verapamil and nicardipine enhanced ADM cytotoxicity in H69AR cells slightly but these effects were not dose-dependent. These results demonstrate that the reversal of drug resistance by verapamil and other calcium antagonists in a dose-dependent fashion is not an invariable property of multidrug-resistant tumour cells.

One of the major reasons for eventual treatment failure in initial responsive tumours is the appearance and proliferation of multidrug-resistant (MDR) tumour cells. A large number of mammalian cell lines have been described which exhibit the MDR phenotype and thus provide a model for the study of this clinical problem. Even though selected with a single agent, these cell lines are resistant to a wide range of chemically and functionally unrelated drugs. A variety of biochemical changes in MDR cells have been described but the most consistent alteration has been the increased expression of a high molecular weight plasma membrane glycoprotein (Gerlach *et al.*, 1986). This protein was first described by Ling & Thompson (1974) in Chinese hamster ovary (CHO) cells and has been termed P-glycoprotein. Considerable evidence suggests that this protein is responsible for increased efflux of drug from MDR cells (Gros *et al.*, 1986). Enhanced P-glycoprotein expression has been detected in tumour samples from MDR patients with ovarian carcinoma (Bell *et al.*, 1985), leukaemias (Ma *et al.*, 1987) and fibrosarcomas (Gerlach *et al.*, 1987) indicating that the phenomenon occurs in humans as well. While P-glycoprotein has clearly been shown to play an important role in MDR, it is unlikely that it is solely responsible for MDR in all tumour cells (Kaye & Merry, 1985). In fact, several human cell lines displaying an MDR phenotype have recently been described in which no evidence of enhanced P-glycoprotein expression can be found by immunoassay or RNA blotting methods (Slovak *et al.*, 1988; Mirski *et al.*, 1987; Marsh & Center, 1987; Trent *et al.*, 1988).

Tsuruo *et al.* (1981) were the first to report that verapamil (VP), a calcium channel blocker, could reverse MDR *in vitro*. A variety of compounds have since been identified which enhance drug sensitivity *in vitro* and are often referred to as 'chemosensitisers'. Included in this group are such diverse compounds as nicardipine (Ramu *et al.*, 1984*c*), trifluoperazine (Ganapathi & Grabowski, 1983), tamoxifen (Ramu *et al.*, 1984*b*), quinidine (Tsuruo *et al.*, 1984), reserpine (Inaba *et al.*, 1981), chloroquine (Zamora & Beck, 1986), propranolol (Shiraishi *et al.*, 1986), perhexiline maleate (Ramu *et al.*, 1984*a*), synthetic isoprenoids (Yamaguchi *et al.*, 1986), cyclosporin A (Slater *et al.*, 1986) and local anaesthetics (Chlebowski *et al.*, 1982). While most, if not all, of these compounds affect calcium metabolism, it is generally agreed that the ability of these agents to enhance chemosensitivity results from interactions with a membrane transport system and not via modulation of calcium fluxes (Ramu *et al.*, 1984*a,c*; Kessel & Wilberding, 1984). Despite the fact that the mode of action of chemosensitisers is not yet understood (Hindenburg *et al.*, 1987; Harker *et al.*, 1986; Ganapathi *et al.*, 1986), these chemicals are being evaluated in clinical trials (Ozols *et al.*, 1987; Dalton *et al.*, 1987, 1989; Tormey *et al.*, 1982).

Many of the cell lines in which reversal of resistance has been demonstrated have also been shown to express enhanced levels of P-glycoprotein (Zamora & Beck, 1986; Hamada *et al.*, 1987; Tsuruo *et al.*, 1981). The present study was undertaken to determine whether or not adriamycin (ADM) resistance could be pharmacologically reversed in two MDR human tumour cell lines which lack elevated P-glycoprotein.

### Materials and methods

### *Drugs*

Chloroquine diphosphate, clomiphene citrate, nicardipine, perhexiline maleate, tamoxifen citrate, trifluoperazine dihydrochloride, prenylamine, verapamil hydrochloride and MTT [3-(4,5-dimethylthiazol-2-yl)-2,5-diphenyl-tetrazolium bromide] were obtained from Sigma Chemical Co. (St Louis, MO, USA). Adriamycin (Doxorubicin, HCl; ADM) was obtained from Adria Laboratories (Burlington, Ontario) through the Kingston Cancer Clinic pharmacy.

### *Cell culture*

The human small cell lung cancer (SCLC) cell line NCI-H69 (H69) was kindly provided by Dr J. Minna (National Cancer Institute, Bethesda, MD, USA). A MDR variant of H69, designated H69AR, was established by culturing the sensitive cells in increasing concentrations of ADM and has been described previously (Mirski *et al.*, 1987). These cell lines were maintained in RPMI 1640 medium (GIBCO) supple-

Correspondence: S.P.C. Cole.
Received 28 March 1988; and in revised form, 24 August 1988.

mented with 5% heat-inactivated fetal bovine serum (FBS) and 4 mM L-glutamine. H69AR was challenged biweekly with 0.6 $\mu$M ADM.

The human fibrosarcoma cell line HT1080 and the derivation of its MDR variant HT1080/DR4 are described elsewhere (Slovak *et al.*, 1988). These cell lines were maintained in minimal essential medium (GIBCO) supplemented with 10% FBS, 4 mM L-glutamine and non-essential amino acids (GIBCO) (0.1 mM). HT1080/DR4 cells were cultured in 0.2 $\mu$M ADM at all times except for the 48 h before drug additions when the medium was replaced with that containing no ADM. All cell lines were cultured at 37°C under 5% $CO_2$. Cultures were checked for *Mycoplasma* contamination using the 4′,6-diamidino-2-phenylindole DNA-binding method (Russell *et al.*, 1975) and found to be negative.

*MTT assay*

Chemosensitivity was determined using the MTT assay (Mosmann, 1983; Cole, 1986; Mirski *et al.*, 1987). The human fibrosarcoma cell lines were harvested by incubation in citrated phosphate-buffered saline (PBS) for 10 min at 37°C, while H69 and H69AR cells were harvested by centrifugation. After resuspension in fresh medium, the cells were plated in a volume of 100 $\mu$l at $5 \times 10^3$ cells per well for HT1080 and HT1080/DR4 and $2.5 \times 10^4$ cells per well for H69 and H69AR in 96-well plates. These cell densities were chosen because they allowed exponential growth throughout a five-day assay period. The plates were incubated at 37°C overnight and then ADM and chemosensitisers were added in a total volume of 100 $\mu$l and the plate incubated at 37°C for a further five days.

The chemosensitisers were dissolved in methanol, except for trifluoperazine which was dissolved in dimethyl sulphoxide and chloroquine which was dissolved in tissue culture medium. Stock solutions were diluted in RPMI 1640 5% FBS medium as required and were prepared freshly for each experiment. Three hours before the end of the drug exposure time, 100 $\mu$l of medium was removed from each well, 25 $\mu$l of MTT (2 mg ml$^{-1}$ in PBS) were added and the plate incubated at 37°C for an additional 3 hours. One hundred $\mu$l of 1 N HCl:isopropanol (1:24) was added to each well followed by thorough mixing with a multichannel pipette and a further 1 h incubation at 37°C to aid solubilisation of the formazan crystals. The absorbance at 570 nm was determined using a Dynatech MR600 microplate reader. Within each experiment, determinations were done in quadruplicate and each drug was tested in two or more separate experiments. Controls consisted of wells with no cells and wells with cells plus vehicle (baseline). In the combination experiments, wells with cells plus chemosensitiser but no ADM were also included as controls.

Results are expressed as a percentage of the baseline absorbance at 570 nm and an $ID_{50}$ was defined as the dose of drug which reduced absorbance to 50% of control values. Only those combination experiments in which the chemosensitiser alone reduced the baseline absorbance by less than 20% are reported. An ADM dose–response curve was determined in each experiment. Throughout these series of experiments the relative resistance of H69AR cells to ADM ranged from 20 to 141-fold while the relative resistance of HT1080/DR4 cells ranged from 45 to 333-fold. The mean relative resistance ($\pm$s.e.m.) of H69AR and HT1080/DR4 cells was $66 \pm 12$ ($n = 12$) and $88 \pm 21$ ($n = 14$), respectively. In some experiments, low doses of ADM appeared to cause a small (20%) stimulatory effect on the growth of H69AR cells. This effect was not seen with the other cell lines. The basis of this stimulation is unknown but may be similar to that described by Grace *et al.* (1987).

### Results

Eight drugs were tested for their ability to reverse ADM cytotoxicity in two human tumour cell lines (HT1080 and H69) and their-multidrug-resistant variants which do not overexpress P-glycoprotein (HT1080/DR4 and H69AR). These drugs included calcium channel blockers (VP, nicardipine, perhexiline maleate), calmodulin inhibitors (trifluoperazine, prenylamine), anti-oestrogens (clomiphene, tamoxifen) and the anti-malarial agent, chloroquine.

In the first series of experiments, dose–response curves were determined for the chemosensitisers. The results for HT1080 and HT1080/DR4 cells are shown in Figure 1 and the results for H69 and H69AR cells are shown in Figure 2. From these experiments it was determined that the maximal non-toxic doses of chemosensitisers for HT1080/DR4 cells were VP 10 $\mu$M, chloroquine 10 $\mu$M, trifluoperazine 3 $\mu$M, clomiphene 3 $\mu$M, tamoxifen 1 $\mu$M, prenylamine 1 $\mu$M, nicardipine 3 $\mu$M and perhexiline maleate 1 $\mu$M. HT1080 cells were equally sensitive to the chemosensitisers, except for nicardipine which was slightly more cytotoxic to HT1080/DR4 cells than HT1080 cells (Figure 1). The maximal non-toxic doses for H69AR cells were VP 10 $\mu$M, trifluoperazine 1 $\mu$M, clomiphene 1 $\mu$M, tamoxifen 3 $\mu$M, prenylamine 1 $\mu$M, nicardipine 3 $\mu$M and perhexiline maleate 1 $\mu$M. H69 cells were equally sensitive to all drugs except chloroquine and clomiphene. H69 cells were slightly more sensitive to clomiphene than H69AR cells, while H69AR cells exhibited considerable cross-resistance to chloroquine (Figure 2).

In the second series of experiments, the combined effect of ADM and chemosensitiser on the viability of the fibrosarcoma cell lines was determined at non-toxic doses of chemosensitiser. The results are shown in Table I. Of the eight drugs tested, only VP had any significant chemosensitising effect on HT1080/DR4 cells and appeared to do so in a dose-dependent manner. However, this effect was modest. Thus 10 $\mu$M and 1 $\mu$M VP reduced the $IC_{50}$ of ADM 7.4-fold and 4.2-fold, respectively, in HT1080/DR4 cells. The effect in HT1080 cells was not significant. These results are in excellent agreement with those obtained independently by Slovak *et al.* (1988) using a slightly modified MTT assay (Carmichael *et al.*, 1987).

In the last series of experiments, the combined effect of ADM and chemosensitiser on the viability of the SCLC cell lines was determined. The results are shown in Table II. None of the drugs enhanced the cytotoxic effects of ADM on H69 cells. Chloroquine, trifluoperazine, clomiphene and tamoxifen did not enhance the sensitivity of H69AR cells while VP, prenylamine, nicardipine and perhexiline maleate caused a very modest (maximum 4.9-fold) reversal of resistance in H69AR cells. However, in no case was this effect dose-dependent.

### Discussion

The development of drug resistance in cancer patients is widely recognised as a major impediment to successful chemotherapy. Thus the discovery that the calcium channel blocker VP could reverse MDR in an experimental system was an important finding since it has provided a strategy for treating this clinical problem.

Many structurally diverse chemosensitisers in addition to VP have been identified in experimental systems. However, no single agent has been demonstrated to be clearly superior to others nor has the mode of action of these compounds been elucidated. In fact, current evidence suggests that chemosensitising agents may act by different mechanisms (Kessel, 1986). Despite this lack of information, VP has made its way into several clinical trials. Ozols *et al.* (1987) were unable to find any chemosensitising effect of VP for ADM in eight drug resistant ovarian cancer patients. On the other hand, Dalton *et al.* (1987, 1989) have demonstrated a therapeutic benefit of VP for four patients with P-glycoprotein-positive B-cell neoplasms. These findings demonstrate that it is not yet possible to predict the clinical usefulness of these agents in a given group of patients. Furthermore, there is insufficient information to allow the

**Figure 1** Effect of chemosensitisers on the growth of human fibrosarcoma cell lines, HT1080 (○) and multidrug resistant HT1080/DR4 (●) as measured by the MTT assay. Cells were set up at $5 \times 10^3$ cells per well on day 0, drugs added on day 1 and cell viability measured on day 5. Within each experiment, assays were done in quadruplicate. Each point represents the mean of results from two or more individual experiments which varied $\leqslant 10\%$. Error bars represent the s.d. where three or more experiments were performed.

**Figure 2** Effect of chemosensitisers on the growth of small cell lung cancer cell lines, H69 (○) and multidrug resistant H69AR (●) as measured by the MTT assay. Cells were set up at $2.5 \times 10^4$ cells per well on day 0, drugs added on day 1 and cell viability measured on day 5. Within each experiment, assays were done in quadruplicate. Each point represents the mean of results from two or more individual experiments which varied $\leqslant 10\%$. Error bars represent the s.d. where three or more experiments were performed.

**Table I** Effect of chemosensitisers on ADM sensitivity of human fibrosarcoma cell lines HT1080 and HT1080/DR4

| *Drug* | *Concentration (μM)* | *Dose modifying factor*[a] *HT1080* | *HT1080/DR4* |
|---|---|---|---|
| Verapamil | 10 | 2.63 (±1.42)[b] | 7.43 (±0.57) |
| | 3 | 2.48 (1.75, 3.20) | 4.21 (4.45, 3.96) |
| | 1 | 1.46 (1.11, 1.80) | 1.42 |
| Chloroquine | 10 | 1.42 | 1.27 |
| | 3 | 1.01 (±0.51) | 1.11 (0.80, 1.41) |
| | 1 | 1.09 (±0.86) | 1.23 (±0.73) |
| Trifluoperazine | 3 | 1.20 (1.26, 1.13) | 1.04 (1.27, 0.80) |
| | 1 | 1.01 (1.13, 0.89) | 1.21 (1.42, 1.00) |
| Clomiphene | 3 | 1.41 (±0.15) | 1.06 (±0.30) |
| | 1 | 1.13 (±0.14) | 1.05 (±0.14) |
| Tamoxifen | 1 | 0.90 (1.00, 0.80) | 1.27 (1.12, 1.42) |
| Prenylamine | 1 | 0.95 (1.00, 0.90) | 0.96 (0.79, 1.12) |
| | 0.3 | 1.01 (1.11, 0.90) | 0.88 (0.63, 1.12) |
| Nicardipine | 3 | 1.25 (1.39, 1.11) | 1.06 (1.00, 1.12) |
| Perhexiline | 1 | 1.50 (1.75, 1.25) | 1.26 (1.25, 1.26) |
| | 0.3 | 1.25 (1.39), 1.11) | 1.00 (1.00, 1.00) |

[a]Dose modifying factor is defined as the $ID_{50}$ ADM: $ID_{50}$ (ADM+chemosensitiser); [b]s.d. given in parentheses where three or more experiments were performed. The results of individual experiments are given where less than three experiments were done. Within each experiment, assays were done in quadruplicate.

**Table II** Effect of chemosensitisers on ADM sensitivity of small cell lung cancer cell lines H69 and H69AR

| *Drug* | *Concentration (μM)* | *Dose modifying factor*[a] *HT69* | *HT69AR* |
|---|---|---|---|
| Verapamil | 10 | 0.91 (0.82, 1.00)[b] | 4.86 (±1.96) |
| | 3 | 1.08 (±0.52) | 1.81 (±1.02) |
| | 1 | 1.24 (±0.70) | 2.42 (±0.79) |
| Chloroquine | 3 | 1.84 (2.54, 1.13) | 1.34 (1.41, 1.26) |
| | 0.3 | 0.94 (±0.34) | 2.81 (±2.48) |
| Trifluoperazine | 1 | 2.32 (2.86, 1.78) | 1.63 (1.25, 2.00) |
| | 0.3 | 0.80 (±0.41) | 1.27 (±0.59) |
| Clomiphene | 1 | 1.69 (1.80, 1.58) | 1.74 (±0.70) |
| | 0.3 | 0.85 (±0.20) | 0.99 (±0.59) |
| Tamoxifen | 3 | 1.13 | 1.23 (±0.35) |
| | 1 | 1.49 (±1.13) | 2.30 (1.42, 3.17) |
| Prenylamine | 1 | 1.63 (±0.52) | 2.59 (±0.72) |
| | 0.3 | 1.85 (±1.50) | 3.33 (±1.64) |
| Nicardipine | 3 | 1.09 (±0.18) | 4.59 (5.61, 3.56) |
| | 1 | 1.41 (1.42, 1.40) | 4.22 (3.98, 4.48) |
| Perhexiline | 1 | 0.94 (±0.58) | 2.44 (±1.02) |
| | 0.3 | 1.32 (0.63, 2.00) | 3.93 (5.04, 2.81) |

[a]Dose modifying factor is defined as the $ID_{50}$ ADM: $ID_{50}$ (ADM+chemosensitiser); [b]s.d. given in parentheses where three or more experiments were performed. The results of individual experiments are given where less than three experiments were done. Within each experiment, assays were done in quadruplicate.

rational selection of the optimal chemosensitiser for each group.

There are many possible explanations for the contrasting results reported by Ozols *et al.* (1987) and Dalton *et al.* (1987), including methodological differences. It is also possible that only certain tumour types respond to the effects of chemosensitising agents. Another possibility is that only drug resistance associated with P-glycoprotein is susceptible to reversal by VP and other compounds (Croop *et al.*, 1987). The results of the present study support this latter idea since, of eight different agents tested, only VP (in the case of HT1080/DR4 cells) and VP and nicardipine (in the case of H69AR cells) had any ability to enhance ADM cytotoxicity. In all instances, the enhancing effect was modest and, in the H69AR cells, the effects were not dose-dependent, suggesting a limited potential for calcium antagonists to reverse resistance in these cell lines. Neither the HT1080/DR4 cell line nor the H69AR cell line overexpress P-glycoprotein (Trent *et al.*, 1988). Further support for this idea comes from the study of Beck *et al.* (1987) who found that VP did not reverse resistance in an 'atypical' (non-P-glycoprotein-containing) MDR human leukaemic cell line.

The results obtained in the present study with SCLC cell line H69AR contrast with those of Twentyman *et al.* (1987). These investigators were able to show a clear dose-dependent enhancement of ADM sensitivity by VP in a MDR variant of NCI-H69, H69/LX4, derived independently from us. However, it is noteworthy that H69/LX4 cells express elevated levels of P-glycoprotein (J.J. Reeve & P. Twentyman, personal communication). These results support the idea that the presence of elevated P-glycoprotein may be a predictor of response to chemosensitising agents in SCLC and other cancers. More extensive laboratory and clinical studies are necessary to test this hypothesis.

H.F. Downes was a recipient of a Natural Sciences and Engineering Research Council (Canada) summer studentship. M.L. Slovak is a recipient of a Cancer Research Fellowship on behalf of the Ladies Auxiliary of the Veterans of Foreign Wars of the United States. This work was supported by grants to S.P.C. Cole from the MRC (Canada) and the NCI (Canada). We thank Drs W.S. Dalton, J.H. Gerlach, W.J. Mackillop, S.E.L. Mirski, J.M. Trent and P.R. Twentyman for helpful discussions.

### References

BECK, W.T., CIRTAIN, M.C., DANKS, M.K. & 5 others (1987). Pharmacological, molecular, and cytogenetic analysis of 'atypical' multidrug-resistant human leukemic cells. *Cancer Res.*, **47**, 5455.

BELL, D.R., GERLACH, J.H., KARTNER, N., BUICK, R.N. & LING, V. (1985). Detection of p-glycoprotein in ovarian cancer: A molecular marker associated with multidrug resistance. *J. Clin. Oncol.*, **3**, 311.

CARMICHAEL, J., DEGRAFF, W.G., GAZDAR, A.F., MINNA, J.D. & MITCHELL, J.B. (1987). Evaluation of a tetrazolium-based semiautomated colorimetric assay: Assessment of chemosensitivity testing. *Cancer Res.*, **47**, 936.

CHLEBOWSKI, R.T., BLOCK, J.B., CUNDIFF, D. & DIETRICH, M.F. (1982). Doxorubicin cytotoxicity enhanced by local anesthetics in a human melanoma cell line. *Cancer Treat. Rep.*, **66**, 121.

COLE, S.P.C. (1986). Rapid chemosensitivity testing of human lung tumour cells using the MTT assay. *Cancer Chemother. Pharmacol.*, **17**, 259.

CROOP, J.M., GUILD, B.C., GROS, P. & HOUSMAN, D.E. (1987). Genetics of multidrug resistance: Relationship of a cloned gene to the complete multidrug resistant phenotype. *Cancer Res.*, **47**, 5982.

DALTON, W.S., DURIE, B.G.M., SALMON, S.E., GROGAN, T.M., SCHEPER, R.J. & MELTZER, P.S. (1987). Multidrug resistance in multiple myeloma: Detection of P-glycoprotein in clinical specimens and reversal of resistance with verapamil. *Blood*, **70**, 834. (abstract).

DALTON, W.S., GROGAN, T.M., DURIE, B.G.M. & 5 others (1989). Drug resistance in multiple myeloma and non-Hodgkin's lymphoma: Detection of P-glycoprotein and potential circumvention by addition of verapamil to chemotherapy.

GANAPATHI, R. & GRABOWSKI, D. (1983). Enhancement of sensitivity to adriamycin in resistant P388 leukemia by the calmodulin inhibitor trifluoperazine. *Cancer Res.*, **43**, 3696.

GANAPATHI, R., YEN, A., GRABOWSKI, D., SCHMIDT, H., TURINIC, R. & VALENZUELA, R. (1986). Role of the calmodulin inhibitor trifluoperazine on the induction and expression of cell cycle traverse perturbations and cytotoxicity of daunorubicin and doxorubicin (Adriamycin) in doxorubicin-resistant P388 mouse leukaemia cells. *Br. J. Cancer*, **53**, 561.

GERLACH, J.H., BELL, D.R., KARAKOUSIS, C. & 5 others (1987). P-Glycoprotein in human sarcoma: Evidence for multidrug resistance. *J. Clin. Oncol.*, **5**, 1452.

GERLACH, J.H., KARTNER, N., BELL, D.R. & LING, V. (1986). Multidrug resistance. *Cancer Surveys*, **5**, 25.

GRACE, T., KIMBERLY, P., HACHER, M. & TRITTON, T. (1987). Stimulation of growth by adriamycin (abstract). *Proc. Am. Assoc. Cancer Res.*, **28**, 266.

GROS, P., BEN NERIAH, Y., CROOP, J.M. & HOUSMAN, D.E. (1986). Isolation and expression of a complementary DNA that confers multidrug resistance, *Nature*, **323**, 728.

HAMADA, H., HAGIWARA, K-I., NAKAJIMA, T. & TSURUO, T. (1987). Phosphorylation of the $M_r$ 170,000 to 180,000 glycoprotein specific to multidrug-resistant tumour cells: Effects of verapamil, trifluoperazine, and phorbol esters. *Cancer Res.*, **47**, 2860.

HARKER, W.G., BAUER, D., ETIZ, B.B., NEWMAN, R.A. & SIKIC, B.I. (1986). Verapamil-mediated sensitization of doxorubicin-selected pleiotropic resistance in human sarcoma cells: selectivity for drugs which produce DNA scission. *Cancer Res.*, **46**, 2369.

HINDENBURG, A.A., BAKER, M.A., GLEYZER, E., STEWART, V.J., CASE, N. & TAUB, R.N. (1987). Effect of verapamil and other agents on the distribution of anthracyclines and on reversal of drug resistance. *Cancer Res.*, **47**, 1421.

INABA, M., FUJIKURA, R., TSUKAGOSHI, S. & SAKURAI, Y. (1981). Restored *in vitro* sensitivity of adriamycin- and vincristine-resistant P388 leukemia with reserpine. *Biochem. Pharmacol.*, **30**, 2191.

KAYE, S. & MERRY, S. (1985). Tumour cell resistance to anthracyclines – a review. *Cancer Chemother. Pharmacol.*, **14**, 96.

KESSEL, D. (1986). Interactions among membrane transport systems: Anthracyclines, calcium antagonists and anti-estrogens. *Biochem. Pharmacol.*, **35**, 2825.

KESSEL, D. & WILBERDING, C. (1984). Mode of action of calcium antagonists which alter anthracycline resistance. *Biochem. Pharmacol.*, **33**, 1157.

LING, V. & THOMPSON, L.H. (1974). Reduced permeability in CHO cells as a mechanism of resistance to colchicine. *J. Cell. Physiol.*, **83**, 103.

MA, D.D.F., DAVEY, R.A., HARMAN, D.H. & 5 others (1987). Detection of a multidrug resistant phenotype in acute non-lymphoblastic leukaemia. *Lancet*, **i**, 135.

MARSH, W. & CENTER, M.S. (1987). Adriamycin resistance in HL-60 cells and accompanying modification of a surface membrane protein contained in drug-sensitive cells. *Cancer Res.*, **47**, 5080.

MIRSKI, S.E.L., GERLACH, J.H. & COLE, S.P.C. (1987). Multidrug resistance in a human small cell lung cancer cell line selected in adriamycin. *Cancer Res.*, **47**, 2594.

MOSMANN, T. (1983). Rapid colorimetric assay for cellular growth and survival: Application to proliferation and cytotoxicity assays. *J. Immunol. Methods*, **65**, 55.

OZOLS, R., CUNNION, R.E., KLECKER, R.W. & 4 others (1987). Verapamil and adriamycin in the treatment of drug-resistant ovarian cancer patients. *J. Clin. Oncol.*, **5**, 641.

RAMU, A., FUKS, Z., GATT, S. & GLAUBIGER, D. (1984*a*). Reversal of acquired resistance to doxorubicin in P388 murine leukemia cells by perhexiline maleate. *Cancer Res.*, **44**, 144.

RAMU, A., GLAUBIGER, D. & FUKS, Z. (1984*b*). Reversal of acquired resistance to doxorubicin in P388 murine leukemia cells by tamoxifen and other triparanol analogues. *Cancer Res.*, **44**, 4392.

RAMU, A., SPANIER, R., RAHAMIMOFF, H. & FUKS, Z. (1984*c*). Restoration of doxorubicin responsiveness in doxorubicin-resistant P388 murine leukaemia cells. *Br. J. Cancer*, **50**, 501.

RUSSELL, W.C., NEWMAN, C. & WILLIAMSON, D.H. (1975). A simple cytochemical technique for demonstration of DNA in cells infected with mycoplasmas and viruses. *Nature*, **253**, 461.

SHIRAISHI, N., AKIYAMA, S-I., KOBAYASHI, M. & KUWANO, M. (1986). Lysosomotropic agents reverse multiple drug resistance in human cancer cells. *Cancer Lett.*, **30**, 251.

SLATER, L.M., SWEET, P., STUPECKY, M., WETZEL, M.W. & GUPTA, S. (1986). Cyclosporin A corrects daunorubicin resistance in Ehrlich ascites carcinoma. *Br. J. Cancer*, **54**, 235.

SLOVAK, M.L., HOELTGE, G.A., DALTON, W.S. & TRENT, J.M. (1988). Pharmacologic and biologic evidence for differing mechanisms of doxorubicin resistance in two human tumor cell lines. *Cancer Res.*, **48**, 2793.

TORMEY, D.C., FALKSON, G., CROWLEY, J., FALKSON, H.C., VOELKEL, J. & DAVIS, T.E. (1982). Dibromodulcitol and adriamycin ± tamoxifen in advanced breast cancer. *Am. J. Clin. Oncol.*, **5**, 33.

TRENT, J.M., MELTZER, P.S., SLOVAK, M.L. & 4 others (1988). Cytogenetic and molecular biologic alterations associated with anthracycline resistance. In *Mechanisms of Drug Resistance in Neoplastic Cells*, Woolley, P.V. & Tew, K.D. (eds) p. 259. Academic Press: New York.

TSURUO, T., IIDA, H., KITATANI, Y., YOKOTA, K., TSUKAGOSHI, S. & SAKURAI, Y. (1984). Effects of quinidine and related compounds on cytotoxicity and cellular accumulation of vincristine and adriamycin in drug-resistant tumor cells. *Cancer Res.*, **44**, 4303.

TSURUO, T., IIDA, H., TSUKAGOSHI, S. & SAKURAI, Y. (1981). Overcoming of vincristine resistance in P388 leukemia *in vivo* and *in vitro* through enhanced cytotoxicity of vincristine and vinblastine by verapamil. *Cancer Res.*, **41**, 1967.

TWENTYMAN, P.R., FOX, N.E. & BLEEHEN, N.M. (1987). Drug resistance in human lung cancer cell lines: Cross-resistance studies and effects of the calcium transport blocker, verapamil. *Int. J. Rad. Oncol. Biol. Phys.*, **12**, 1355.

YAMAGUCHI, T., NAKAGAWA, M., SHIRAISHI, N. & 5 others (1986). Overcoming drug resistance in cancer cells with synthetic isoprenoids. *J. Natl Cancer Inst.*, **76**, 947.

ZAMORA, J.M. & BECK, W.T. (1986). Chloroquine enhancement of anticancer drug cytotoxicity in multiple drug resistant human leukemic cells. *Biochem. Pharmacol.*, **35**, 4303.